# Data on the effect of hypomyelinating leukodystrophy 6 (HLD6)-associated mutations on the TUBB4A properties

**DOI:** 10.1016/j.dib.2017.02.024

**Published:** 2017-02-16

**Authors:** Yuki Miyamoto, Tomohiro Torii, Kazuko Kawahara, Nanami Hasegawa, Akito Tanoue, Yoichi Seki, Takako Morimoto, Megumi Funakoshi-Tago, Hiroomi Tamura, Keiichi Homma, Masahiro Yamamoto, Junji Yamauchi

**Affiliations:** a Laboratory of Molecular Neuroscience and Neurology, School of Life Sciences, Tokyo University of Pharmacy and Life Sciences, Hachioji, Tokyo 192-0355, Japan; b Department of Pharmacology, National Research Institute for Child Health and Development, Setagaya, Tokyo 157-8535, Japan; c Department of Neuroscience, Baylor College of Medicine, Houston, TX 77030, USA; d Department of Hygienic Chemistry, Faculty of Pharmacy, Keio University, Minato, Tokyo 105-8512, Japan; e Department of Life Science and Informatics, Maebashi Institute of Technology, Maebashishi, Gunma 371-0816, Japan; f Tsumura Research Laboratories, Tsumura & Co., Inashiki, Ibaraki 200-1192, Japan

**Keywords:** HLD6, TUBB4A, Tubulin, Disease-associated mutation, Biochemical property

## Abstract

Hypomyelinating leukodystrophy (HLD) is genetic demyelinating or dysmyelinating disease and is associated with at least 13 responsible genes. The mutations seem likely cause the functional deficiency of their gene products. HLD4- and HLD5-associated HSPD1 and FAM126A mutations affect biochemical properties of the gene products (Miyamoto et al. (2015,2014) [[1], [2]]). Herein we provide the data regarding the effects of HLD6-associated tubulin beta 4A (TUBB4A) mutations on the properties.

**Specifications table**

**Table:** 

Subject area	Biology
More specific subject area	Molecular and cellular neurobiology, Molecular and cellular neurology
Type of data	Figure
How data was acquired	GFP-fluorescence, immunoblotting,
Data format	Raw data, analyzed data
Experimental factors	Cos-7 cells were transfected with the plasmid encoding the TUBB4A mutant or the wild type and used for experiments.
Experimental features	GFP-fluorescence analysis, immunoprecipitation analysis
Data source location	Laboratory of Molecular Neuroscience and Neurology, Tokyo University of Pharmacy and Life Sciences, Tokyo, Japan
Data accessibility	Data is available with this article

**Value of the data**

• This data set is of value to the scientific community to need the information for the biochemical and cellular effect of a genetic leukodystrophy-associated mutation on the gene product.
• The data can provide the method to examine change of the property by a leukodystrophy-associated mutation.
• The data allow us to promote how a leukodystrophy-associated mutation affects the property.

## 1 Data

The data shared in this article provide GFP-fluorescent analyses of HLD6-associated TUBB4A mutant proteins. The data also provide immunoprecipitation analyses of HLD6-associated TUBB4A mutant proteins with tubulin alpha.

## 2 Experimental design, materials and methods

### 2.1 Data from HLD6-associated TUBB4A mutant proteins

**Fig. 1:**
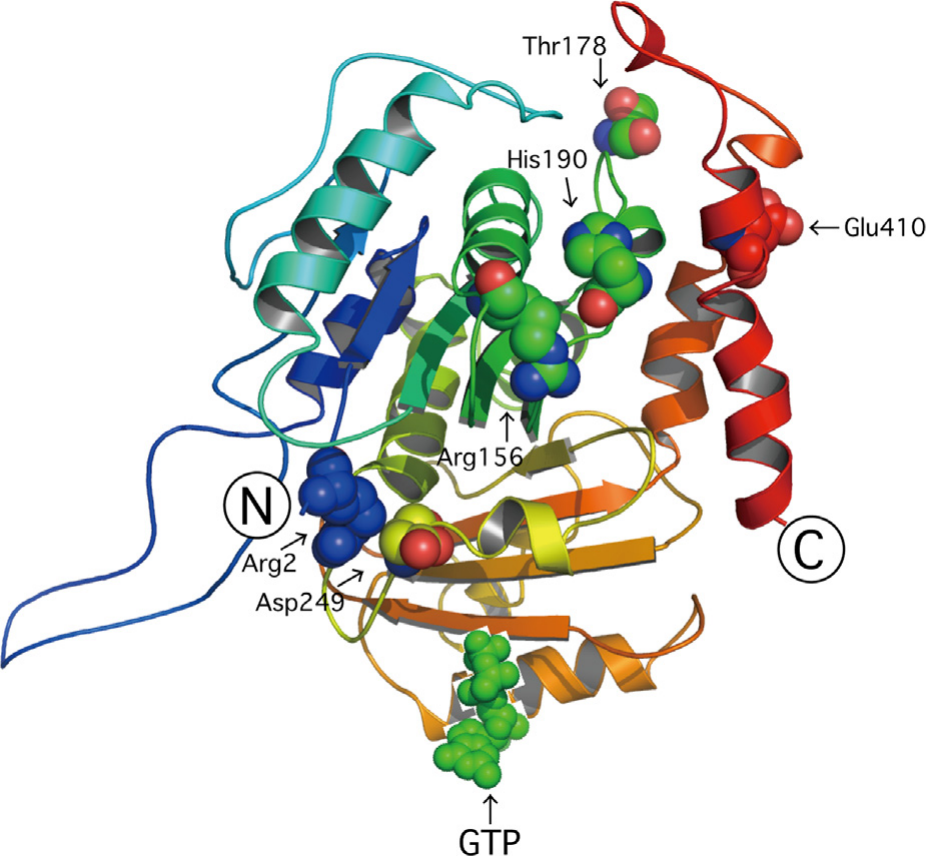
Predicted human TUBB4A 3D structure. The predicted 3D model of human TUBB4A was generated on the basis of the structure of bovine TUBB4A. The amino acid positions of HLD6-associated mutations, as well as N- and C-terminal positions, are shown.

**Fig. 2:**
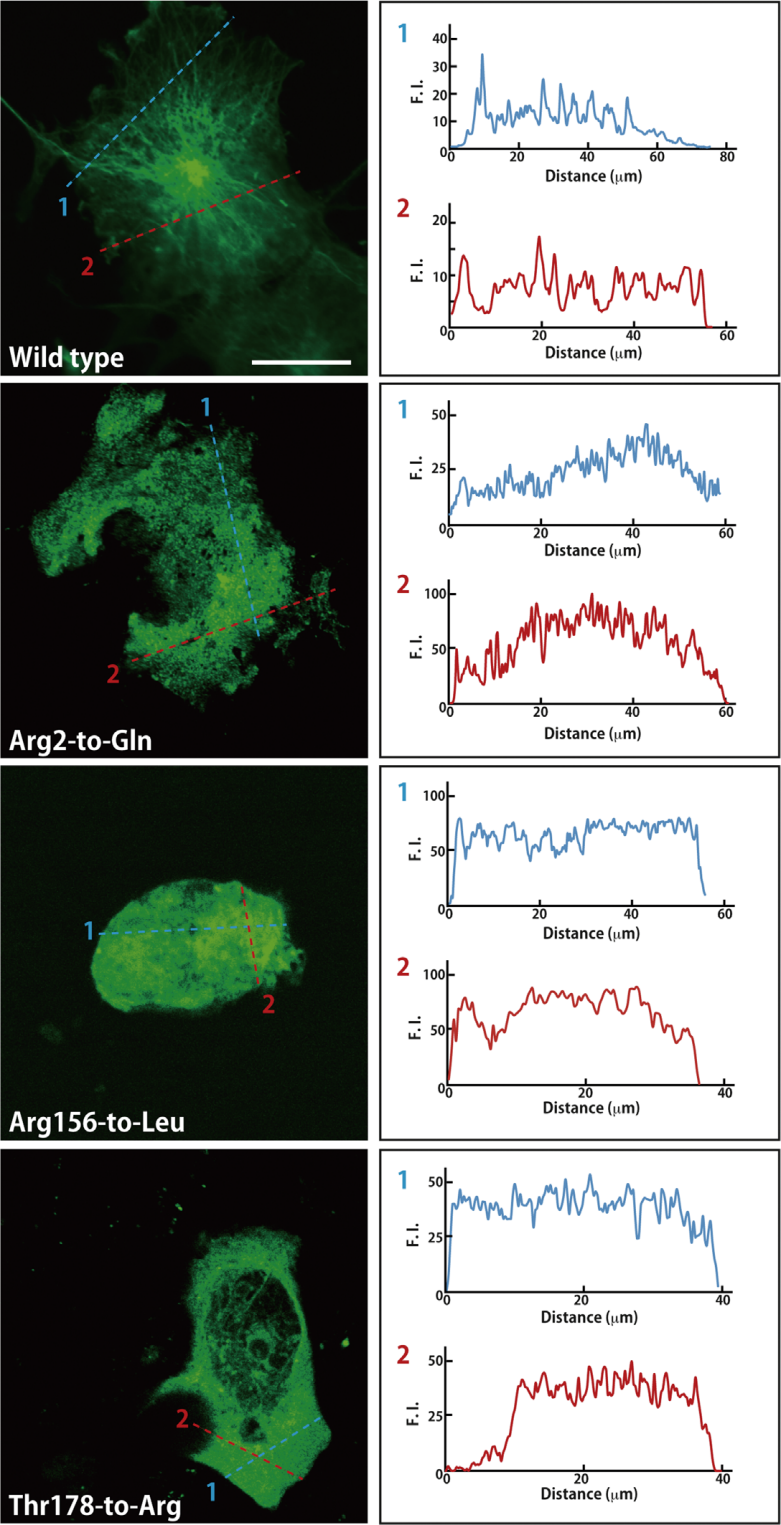
Fluorescent images of the wild type TUBB4A and HLD6-assocated mutants (Arg2-to-Glu, Arg156-to-Leu, and Thr178-to-Arg). Cells were transfected with the plasmids encoding GFP-tagged TUBB4A and the mutants. Representative images (left panels) of GFP-tagged, wild type TUBB4A (with fiber structures) and mutants (without fiber structures) are shown. Fluorescence intensity of lanes 1 (blue) and 2 (red) is also shown in right panels. Scale bar indicates 10 μm.

**Fig. 3:**
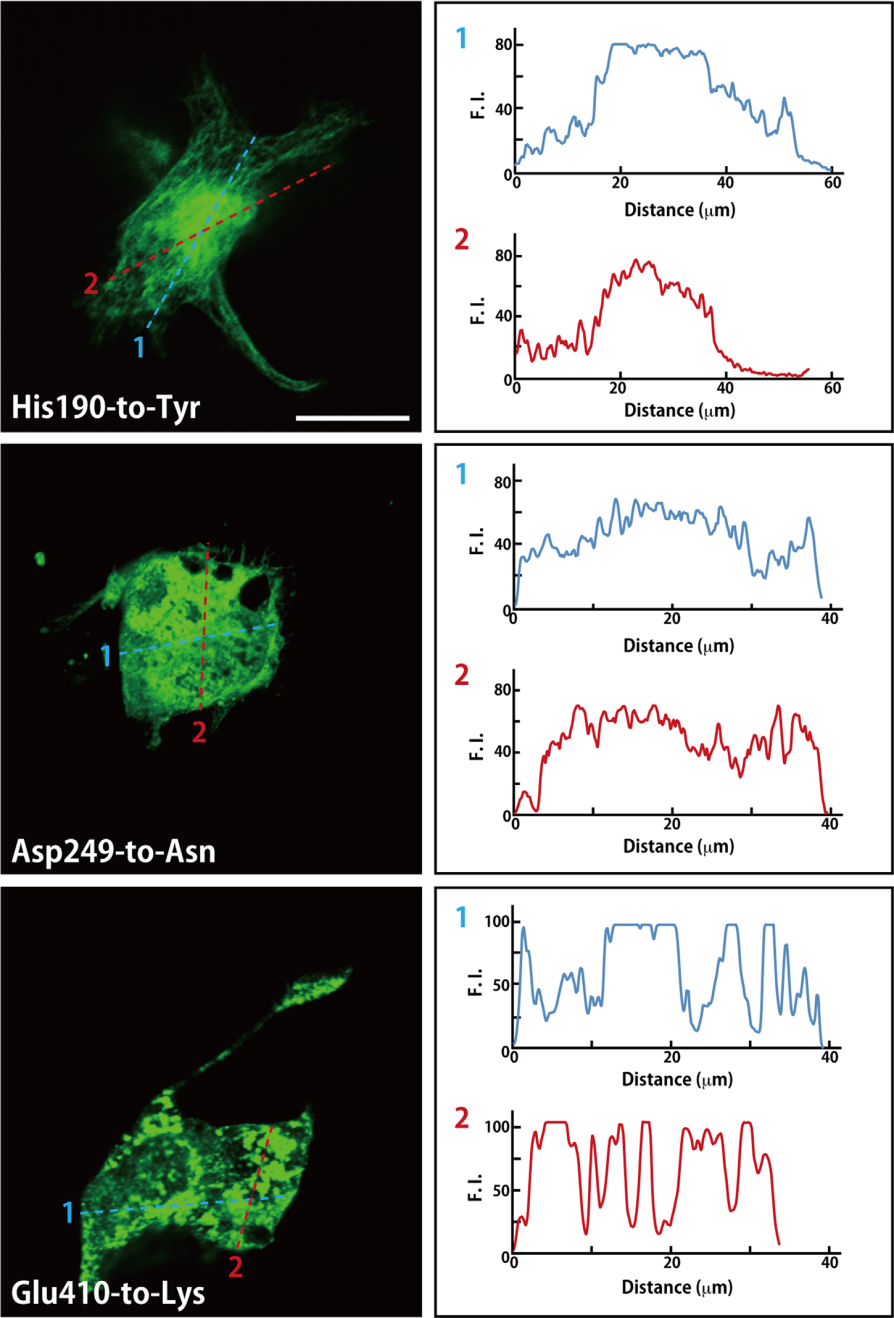
Fluorescent images of HLD6-assocated TUBB4A mutants (His190-to-Tyr, Asp249-to-Asn, and Glu410-to-Lys). Cells were transfected with the plasmids encoding GFP-tagged TUBB4A mutants. Representative images (left panels) of GFP-tagged, mutated TUBB4A are shown in the figure. Fluorescence intensity of lanes 1 (blue) and 2 (red) is also shown in right panels. Scale bar indicates 10 μm.

**Fig. 4:**
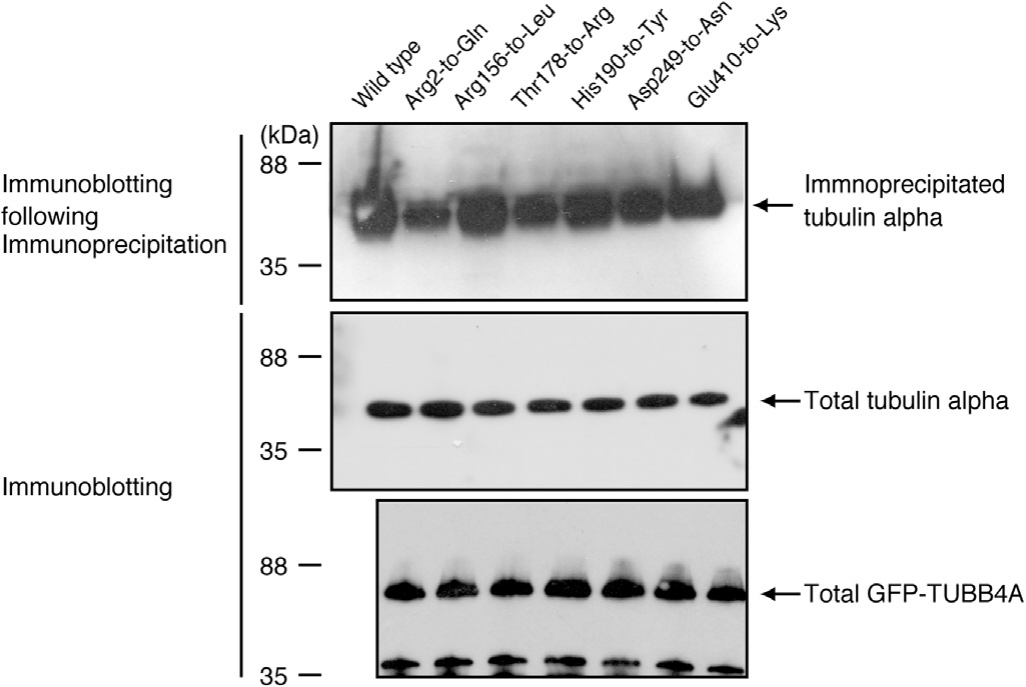
Co-imunoprecipitation of TUBB4A with tubulin alpha. Cells were transfected with the plasmids encoding GFP-tagged TUBB4A and the mutants and lysed. The immunoprecipitates with an anti-GFP antibody were immunoblotted with an anti-tubulin alpha antibody. Total tubulin alpha and GFP-tagged TUBB4A proteins are also shown.

The amino acid positions (Arg2-to-Glu, Arg156-to-Leu, Thr178-to-Arg, His190-to-Tyr, Asp249-to-Asn, and Glu410-to-Lys) of HLD6-associated mutations in TUBB4A are shown in the predicted human TUBB4A׳s 3D model (Fig. 1). All TUBB4A mutants, in GFP-fluorescent images, failed to have the ability to form fiber structure characteristic of the tubulin cytoskeletal protein, being compared with the wild type (Fig. 2, Fig. 3). On the other hand, in co-precipitation experiments of tubulin alpha (tubulin beta binding partner) with TUBB4A mutants, only the Arg2-to-Glu and Thr178-to-Arg mutants showed a weak ability to interact with tubulin alpha (Fig. 4).

### 2.2 Construction of plasmids

Human TUBB4A was amplified from human brain cDNAs (Wako, Osaka, Japan) and ligated into the pEGFP-N3 vector (Takara Bio, Shiga, Japan). All HLD6-associated mutations [3] (OMIN ID: 612438) were produced from pEGFP-N3-human TUBB4A, as the template, using the site-directed mutagenesis kit (Toyobo Life Science, Osaka, Japan), according to the manufacturers׳ instruction. All DNA sequences were confirmed by sequencing (Fasmac, Kanagawa, Japan).

### 2.3 Cell culture and transfection

Cos-7cells (Human Health Science Research Resources Bank, Osaka, Japan) were cultured on cell culture dishes (Greiner, Oberösterreich, Germany) in DMEM containing 10% heat-inactivated FBS and PenStrep (Thermo Fisher Scientific, Waltham, MA, USA) in 5% CO_2_ at 37 °C. Cells were transfected with the plasmids using the Lipofectamine LTX Plus transfection kit (Thermo Fisher Scientific), according to the manufacturers׳ instruction. The medium was replaced 4 h after transfection and used for experiments 48 h after transfection.

### 2.4 GFP-fluorescence

Cells on coverslips were fixed with 100% cold methanol for observation of the tubulin fiber structure. Cells were blocked with the Blocking One reagent (Nacalai Tesque, Kyoto, Japan). The coverslips on a slide glass were mounted with the Vectashield reagent (Vector Laboratories, Burlingame, CA, USA). The fluorescent images were collected with an IX81 microscope system (Olympus, Tokyo, Japan) equipped with a laser-scanning FV500 or FV1000D (Olympus) and analyzed with Fluoview software (Olympus) and Image J software (URL: https://imagej.nih.gov/ij/) [4]. At least three experiments were carried out under each condition and the representative photographs are shown in the figures.

### 2.5 Immunoprecipitation and immunoblotting

Cells were lysed in lysis buffer (50 mM HEPES-NaOH, pH 7.5, 150 mM NaCl, 20 mM MgCl_2_, 1 mM dithiothreitol, 1 mM phenylmethane sulfonylfluoride, 1 μg/ml leupeptin, 1 mM EDTA, 1 mM Na_3_VO_4_, 10 mM NaF, and 0.5% NP-40) and centrifuged as described [1], [2]. The supernatants were mixed with protein G resin that was preadsorbed with a polyclonal anti-GFP antibody (MBL, Aichi, Japan). The immunoprecipitates were washed with lysis buffer and denatured. The samples were separated on SDS–polyacrylamide gels. The electrophoretically separated proteins were transferred to PVDF membranes, blocked with the Blocking One reagent, and immunoblotted using primary antibodies (a monoclonal tubulin alpha antibody [MBL] or a monoclonal anti-GFP antibody [MBL]), followed by peroxidase-conjugated secondary antibodies (Nacalai Tesque). The bound antibodies were detected using the ImmunoStar Zeta kit (Wako). The bands were captured using UN-SCAN-IT Gel software (Silk Scientific, Orem, UT, USA). At least three experiments were carried out under each condition and the representative photographs are shown in the figures.

### 2.6 Molecular modeling

The model of human TUBB4A was generated on the basis of the structure of bovine TUBB4A (Protein Data Bank ID: 1FFXB) with MAFFTash (URL: http://pdbj.org/mafftash/) and PyMOL (URL: https://www.pymol.org/).
